# The challenges and successes of implementing a sustainable antimicrobial resistance surveillance programme in Nepal

**DOI:** 10.1186/1471-2458-14-269

**Published:** 2014-03-21

**Authors:** Sarala Malla, Shyam Prakash Dumre, Geeta Shakya, Palpasa Kansakar, Bhupraj Rai, Anowar Hossain, Gopinath Balakrish Nair, M John Albert, David Sack, Stephen Baker, Motiur Rahman

**Affiliations:** 1 National Public Health Laboratory, Department of Health Services, Ministry of Health and Population, Kathmandu, Nepal; 2 Faculty of Allied Health Science, Tammasat University, Thailand, Thailand; 3 International Centre for Diarrhoeal Disease Research, Dhaka, Bangladesh; 4 Translational Health Science and Technology Institute, New Delhi, India; 5 Department of Microbiology, Faculty of Medicine, Kuwait University, Jabriya, Kuwait; 6 Department of International Health, Johns Hopkins University Bloomberg School of Public Health, Baltimore, MD, USA; 7 The Hospital for Tropical Diseases, Wellcome Trust Major Overseas Programme, Oxford University Clinical Research Unit, 764 Vo Van Kiet Street, Ward 1, District 5, Ho Chi Minh City, Vietnam

**Keywords:** Antimicrobial resistance surveillance, Nepal, Respiratory pathogens, Enteric pathogens, Sexually transmitted disease pathogens

## Abstract

**Background:**

Antimicrobial resistance (AMR) is a major global public health concern and its surveillance is a fundamental tool for monitoring the development of AMR. In 1998, the Nepalese Ministry of Health (MOH) launched an Infectious Disease (ID) programme. The key components of the programme were to establish a surveillance programme for AMR and to develop awareness among physicians regarding AMR and rational drug usage in Nepal.

**Methods:**

An AMR surveillance programme was established and implemented by the Nepalese MOH in partnership with the International Centre for Diarrhoeal Disease Research, Bangladesh (ICDDR, B) from 1998 to 2003. From 2004 to 2012, the programme was integrated and maintained as a core activity of the National Public Health Laboratory (NPHL) and resulted in an increased number of participating laboratories and pathogens brought under surveillance. The main strategies were to build national capacity on isolation, identification and AMR testing of bacterial pathogens, establish laboratory networking and an External Quality Assessment (EQA) programme, promote standardised recording and reporting of results, and to ensure timely analysis and dissemination of data for advocacy and national policy adaptations. The programme was initiated by nine participating laboratories performing AMR surveillance on *Vibrio cholerae, Shigella* spp., *Streptococcus pneumoniae*, *Haemophilus influenzae,* and *Neisseria gonorrhoeae*.

**Results:**

The number of participating laboratories was ultimately increased to 13 and the number of pathogens under surveillance was increased to seven (*Salmonella* spp. was added to the surveillance programme in 2002 and extended spectrum β-lactamase producing *Escherichia coli* in 2011). From 1999 to 2012, data were available on 17,103 bacterial isolates. During the AMR programme, we observed changing trends in serovars/ species for *Salmonella* spp.*, Shigella* spp. *and V. cholerae* and changing AMR trend for all organisms. Notably, *N. gonorrhoeae* isolates demonstrated increasing resistance to ciprofloxacin. Additionally, the performance of the participating laboratories improved as shown by annual EQA data evaluation.

**Conclusions:**

This Nepalese AMR programme continues and serves as a model for sustainable surveillance of AMR monitoring in resource limited settings.

## Background

Antimicrobial resistance (AMR) in bacterial pathogens reduces the effectiveness of antimicrobial agents leading to increased morbidity, mortality and healthcare costs [1]. While the eradication of AMR is not realistic, significant delay in development of AMR can be achieved by rational antimicrobial use [2]. In Nepal, over-the-counter availability, dispensing of antimicrobials without professional consultation, inappropriate usage and the use of antimicrobials with low potency as a result of poor manufacturing and storage conditions or counterfeiting are common, and encourage AMR development [3,4]. Policies and regulations that support appropriate and rational use of antimicrobials are key to the long-term interventions for reducing AMR. Although many global and local strategies and interventions have been developed for containing AMR, surveillance of AMR remains fundamental to combating resistance [5,6]. The World Health Organisation (WHO) recommends that each member-state establish a national surveillance programme of AMR for selected bacteria to monitor drug susceptibility in relevant organisms [7]. In Nepal, there are major challenges in implementing such a programme. This is due to the following factors: lack of appropriately trained personnel, frequent transfer of staff, poor access to good quality reagents, inadequate storage facilities of reagents, frequent power failure, limited funding as a result of competing priorities, and frequent policy changes. The situation has been compounded by political instability and insurgency. In this report, we present our experience of implementing a sustainable national AMR surveillance programme in Nepal.

## Methods

### Local setting

Nepal is a low-income country (as determined by the World bank) with a poorly organised healthcare delivery system, suffering from resource limitations. Political unrest and the Maoist insurgency from 2001 to 2008 affected many sectors of life including sustainable health care delivery. Furthermore, pharmaceutical manufacturing and importation is poorly regulated and drugs, including antimicrobials, are available without prescription, and antimicrobial consumption on sub-therapeutic dose, for sub-optimal duration is common. Additionally, pharmaceuticals are often stored in sub-standard conditions, compromising product quality. The public health care system in Nepal has a limited laboratory capacity for bacterial culture and AMR testing (available only at some regional hospitals).

### Response

In 1998, the Ministry of Health (MOH), Nepal and the United States Agency for International Development (USAID) launched an Infectious Disease (ID) control programme to strengthen national capacity for prevention and control of priority infectious diseases. The goals were to develop a sustainable national surveillance of AMR, develop awareness among physicians regarding AMR and rational drug use, to establish a microbiology quality assurance programme and to systematise record keeping system with data dissemination. The programme in Nepal was implemented by the International Centre for Diarrhoeal Disease Research, Bangladesh (ICDDR, B) Dhaka, Bangladesh in collaboration with Rational Pharmaceutical Management Project (RPM) Arlington, VA, USA. The programme was approved by ICDDR, B research review committee and ethical review committee. The programme started with a participatory planning workshop to build the programme team and to establish a forum for stakeholders (MOH/ Government of Nepal and key organisations -USAID, Nepal and Washington; RPM; USCDC (United States Center for Disease Control and Prevention, Atlanta, Georgia);ICDDR, B; and WHO/Nepal). The workshop focused on development of strategies and approaches of the programme. The programme was designed for selected enteric pathogens (*Vibrio cholerae* and *Shigella* spp*.*), respiratory pathogens (*Streptococcus pneumoniae* and *Haemophilus influenzae*) and sexually transmitted pathogen (*Neisseria gonorrhoeae)*. The National Public Health Laboratory (NPHL) and the Epidemiology and Disease Control Division (EDCD) were identified as the national coordinating laboratory and the national focal point for the programme, respectively.

A baseline assessment of 13 laboratories (in the central, eastern, western regions) was conducted in November 1998 using a pre-defined (location, infrastructure, human resource, instrumentation, shipping of bacterial isolates and willingness to share bacterial isolates and data) assessment tool and nine laboratories were selected to implement the programme. A national capacity building initiative through training and workshops were conducted by ICDDR, B and NPHL at NPHL, ICDDR, B and at the participating laboratories, through 1999–2012. The goal was to develop a pool of trainers to train technologists to compensate for transfer, replacement or separation and to facilitate expansion of trained staff. Training included i) the training of trainers, ii) bacterial isolation and Minimum Inhibitory Concentration (MIC) determination, iii) the isolation, identification and MIC determination of *Salmonella* Typhi, *N. gonorrhoeae*, *S. pneumoniae* and *H. influenzae* and iv) holding annual refresher training workshops. Additionally, consensus workshops and annual meetings were organised during 1999 to 2004. Laboratory testing and procedures were standardised by developing and implementing Standard Operating Procedures (SOPs) for the isolation, identification and AMR testing of the selected pathogens, reporting results, data/record keeping systems, AMR reporting formats, documentation and communication protocols. Each participating laboratory was supported with bacterial culture media, antimicrobial susceptibility discs and antisera at the outset and was encouraged to incorporate the supplies for surveillance of AMR in their annual procurement list. NPHL was also supported with autoclaves, computers and a -86°C freezer. Each laboratory was also supplied with ATCC (American type culture collection) strains for internal quality control (QC).

Each participating laboratory isolated and identified selected pathogens (all consecutive isolates) and performed AMR testing (selected antimicrobial agents for each pathogen using disk diffusion method [8]) and reported data monthly to NPHL and ICDDR, B and sent the isolates to NPHL. At NPHL, all isolates were verified for identification and susceptibility testing and stored at -86°C for future analysis. As very few patients were attending the participating laboratories for sexually transmitted disease (STD) symptoms, isolation of *N. gonorrhoeae* was established at specific STD clinics at Damak and Hetauda. To strengthen partnerships and networking among the laboratories, training visits between NPHL and participating laboratories were organised. Meetings were also organised among participating laboratories to discuss surveillance programme data, technical issues and sharing experiences and challenges in implementation of the programme. Additionally, ICDDR, B and NPHL team routinely visited the laboratories and provided technical assistance to improve the performance. A two-tier system of External Quality Assessment (EQA) programme and inter-laboratory comparison was implemented to ensure quality. The EQA programme included ICDDR, B (1999–2003)/NHPL (2003–2012) sending two isolates to each of the laboratories every three months, and the inter-laboratory comparison included retesting of 10% isolates at ICDDR, B/NPHL (1999–2003) and NHPL (2003–2012). The WHO scoring system was followed for the EQA and confidential evaluation reports were sent to the laboratories.

## Results and discussion

**Figure 1:**
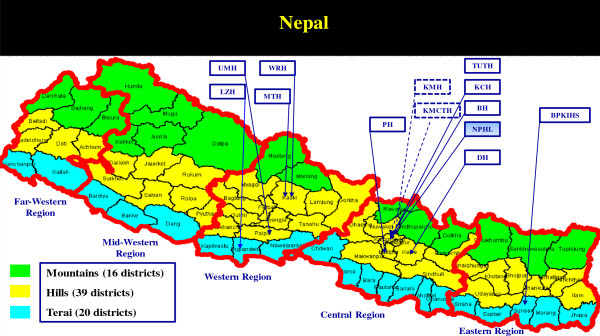
**Geographical distribution and network of laboratories under AMR surveillance programme, Nepal.** NPHL, National Public Health Laboratory; BH, Bir Hospital; PH, Patan Hospital; KCH, Kanti Children’s Hospital; TUTH, Tribhuvan University Teaching Hospital; BPKIHS, B. P. Koirala Institute of Health Sciences; WRH, Western Regional Hospital; MTH, Manipal Teaching Hospital; UMN, United Mission Hospital; LZH, Lumbini Zonal Hospital (included in 2006); DH, Dhulikhel Hospital (included in 2008) and KMH, Kathmandu Model Hospital (included in 2009), KMCTH, Kathmandu Medical College and Teaching Hospital (included in 2010).

The AMR surveillance programme was initiated by a network of nine laboratories in 1999. Lumbini Zonal Hospital (LZH), Butwal, Dhulikhel Hospital (DH), Kabhre, Kathmandu Model Hospital, Katmandu and Kathmandu Medical College and Hospital, Kathmandu, joined the programme in 2006, 2008, 2009 and 2011 respectively (Figure 1). The programme started with five pathogens and *Salmonella* spp. was included in the surveillance in 2002, following an outbreak of enteric fever due to a multiple drug resistant (MDR) *Salmonella* Typhi [9], and extended spectrum β-lactamase (ESBL)- producing *Escherichia coli* was added to the programme in 2009. From 1999 through 2003, ICDDR, B procured and supplied antisera, bacterial culture media and antimicrobial susceptibility discs for the programme. After 2003, the procurement of supplies was integrated into the annual laboratory procurement programme of NPHL.

A total of 10 training workshops and five consensus meetings among participating laboratories were organised during 1999 to 2003, and from 2004 to 2012 annual refresher training was organised among participating laboratories. Training workshops mainly focused on improving the skill of local technicians, technology transfer and creating a pool of local technicians to compensate for transfer and retirements. Consensus meetings were aimed to i) incorporate local innovative ideas generated by local experts (e.g. incorporating salmonella in the surveillance), and ii) encourage participating laboratory management in incorporating surveillance supplies in their respective laboratory procurement plan. Annual meetings were organised among key partners and stakeholders of the programme to i) communicate the progress and the key challenges ii) receive input from all key partners, iii) advocate for increasing the budget for the laboratories and iv) minimise the transfer of surveillance programme staff.

During 1998 to 2003, the ICDDR, B provided technical support to NPHL to function as the national coordination laboratory, and from 2004, NPHL resumed both coordination and technical support responsibilities. Since 2008, NPHL integrated the surveillance of AMR as one of its core activities to ensure the sustainability of the programme.

**Figure 2:**
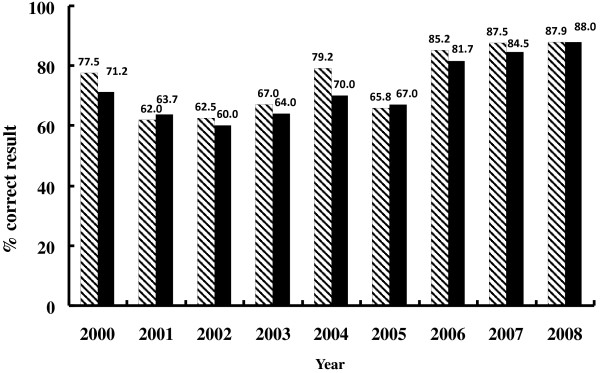
**EQA evaluation of the participating laboratories for organism identification (striped columns) and antimicrobial susceptibility testing (solid columns) during 2000 to 2008.** The EQA programme included sending two isolates to each of the participating laboratories every quarter for organism identification and antimicrobial susceptibility testing.

During 1999–2012, a total of 17,103 bacterial isolates (1,338 *V. cholerae,* 390 *Shigella* spp., 1,875 *S. pneumoniae*, 933 *H. influenzae*, 118 *N. gonorrhoeae,* 11,763 *Salmonella* spp., and 623 *E. coli)* were reported and had corresponding AMR data generated (Table 1) [10-18]. Performance for identification and antimicrobial susceptibility testing of the participating laboratories improved (Figure 2). NPHL, EDCD and the other participating laboratories disseminated the surveillance data through workshops, scientific conferences and journal publications. The AMR surveillance programme contributed to changing the national case management guidelines and national policies for surveillance of several diseases.

**Table 1:** Number of different bacterial pathogens isolated and reported by the AMR surveillance by participating laboratories during 1999 to 2012

**Year**	Vibrio cholerae*	***Shigella *** spp.**	***Streptococcus pneumoniae*** ***	***Haemophilus influenzae*** ****	Neisseria gonorrhoeae*****	***Salmonella *** spp.******	**ESBL *Escherichia coli*** *******	**Total**
1999	61	8	55	2	18	NI	NI	**144**
2000	244	33	155	25	36	NI	NI	**493**
2001	4	40	141	57	21	NI	NI	**263**
2002	25	48	54	115	9	44	NI	**295**
2003	78	36	56	56	22	745	NI	**993**
2004	290	43	83	25	2	510	NI	**953**
2005	62	51	132	60	14	692	NI	**1010**
2006	32	23	92	38	5	1611	NI	**1801**
2007	204	37	120	185	5	1512	NI	**2063**
2008	148	17	189	136	16	1697	NI	**2203**
2009	109	20	213	101	13	1307	14	**1764**
2010	45	9	165	35	7	1525	86	**1872**
2011	1	11	**163**	71	6	**1018**	**76**	**1346**
2012	35	14	257	27	7	1102	447	**1889**
**Total**	**1338**	**390**	**1875**	**933**	**181**	**11763**	**623**	**17103**

Susceptibility of all consecutive isolates collected in the participating laboratories was determined by disk diffusion method [8].

*Susceptibilities for tetracycline, erythromycin, ciprofloxacin, furazolidone, cotrimoxazole, nalidixic acid and ampicillin were determined; a shift in serovars with changing antimicrobial resistance trends was observed for *V. cholerae*, yet the El Tor biotype remained predominant.

**Susceptibilities for ampicillin, ciprofloxacin, cotrimoxazole, nalidixic acid, mecillinam, and azithromycin were determined. *S. dysenteriae* was the predominant species during 1999 – 2004 and *S. flexneri* during 2005 – 2009. *S. dysenteriae* was predominant in eastern Nepal, while *S. flexneri* dominated in the western Nepal. Ciprofloxacin resistant *S. dysenteriae* was common before 2005, which then decreased up to 2007, and re-emerged in 2008. An overall multiresistance rate of 33-75% in *shigella isolates* was found with individual temporal and species variations from *S. dysenteriae* to *S. flexneri*.

***Susceptibilities for penicillin, ampicillin, erythromycin, ciprofloxacin, cotrimoxazole, chloramphenical, and ceftriaxone were determined; *S. pneumoniae* maintained a persistently high level of resistance (58-74%) to cotrimoxazole. Amoxicillin resistance increased to 13% in 2010. One-third of the pneumococcal strains were isolated from children below 15 years with 21% of all isolates from children less than five years of age.

****Susceptibilities for penicillin, ampicillin, amoxi-clav, ciprofloxacin, cotrimoxazole, erythromycin, chloramphenicol, azithromycin and ceftriaxone were determined. Cotrimoxazole resistance remained high (up to 60%) while more than a quarter of the isolates were resistant to at least ampicillin, penicillin and erythromycin. Penicillin resistance reached 100% in 2010.

*****Susceptibilities for penicillin, tetracycline, ciprofloxacin, spectinomycin, azithromycin and ceftriaxone were determined. Prevalence of resistance was high for ciprofloxacin (14-30%) and tetracycline (more than 70%). Ceftriaxone remained 100% susceptible.

******Susceptibilities for ampicillin, tetracycline, ciprofloxacin, cotrimoxazole, nalidixic acid, chloramphenicol, and ceftriaxone were determined. Increasing resistance of *S.* Typhi and *S.* Paratyphi A to nalidixic acid was observed (the resistance rate was higher among *S.* Paratyphi A than among *S.* Typhi. The prevalence of *S.* Paratyphi A increased annually indicating changing epidemiology. Multi drug resistance (MDR-resistance to ampicillin, chloramphenicol and cotrimoxazole) at a time among salmonella isolates declined from 2002 onwards, and newly emerged MDR isolates (resistant to fluoroquinolone [ciprofloxacin, ofloxacin] and nalidixic acid with additional resistance to tetracycline and cotrimoxazole) were identified in 2005, 2006, 2008, 2009 and 2010. MDR phenomenon has increased again from 2009 (7%) and 2010 (8%). Chloromphenicol sensitivity re-emerged (96% in 2010).

*******All isolates exhibited 100% resistance towards quinolones viz. norfloxacin and ofloxacin followed by 99% resistance to ciprofloxacin. ESBL *E.coli* were found to be susceptible to gentamicin (67%) followed by vhloramphenicol(66%) and amikacin(59%).

NI, not included in the surveillance.

The training programme and consensus meeting contributed significantly to national laboratory capacity building, standardisation and use of protocols for selected pathogens, and added new pathogens for surveillance as per local need. The programme also helped NPHL to build capacity for storing the isolates in Nepal and thereby create a sense of ownership of the programme (isolates and data generated through the programme). The programme created a pool of trained technicians who can compensate for transfer and separation of staff and contribute to expansion of programme staff. Stakeholders’ meeting contributed in motivating the MOH, Nepal to increase the resources for laboratories, and incorporating the programme as a core activity of the NPHL and participating laboratories. The programme laid a solid platform for conducting laboratory- based surveillance in Nepal. Annual meetings and dissemination programme helped to establish a forum for discussion on surveillance of AMR in Nepal.

The surveillance of AMR programme in Nepal contributed to some significant policy changes with regard to choice of antimicrobials and vaccination strategies. The national STD case management guidelines in Nepal recommended a change from ciprofloxacin to cefixime as the first-line therapy for the management of uncomplicated gonococcal infection. Also, as more than 20% of pneumococcal isolates were from children below the age of five years, this facilitated evidence-based decision by the MOH to formulate a strategy for pneumococcal vaccination for Nepalese children [15]. Cotrimoxazole has been previously supplied to health posts and sub-health posts for treatment of respiratory infections. As the surveillance programme revealed that most of the *S. pneumoniae* and *H. influenzae* isolates were resistant to cotrimoxazole, the health authorities initiated discussion on suitable alternatives for cotrimoxazole for respiratory infections in Nepal. Ciprofloxacin is no longer considered as the drug of choice for treating salmonella infections, because of reduced susceptibility, even though it appears susceptible *in vitro*. In this example, laboratory technicians were guided to use nalidixic acid resistance as a proxy screening test to determine reduced fluoroquinolone susceptibility [19] and physicians were also advised to interpret the susceptibility results based on this screening test to prevent treatment failure of enteric fever.

## Conclusion

This study illustrates the success of implementing a AMR surveillance programme in a resource poor developing country. To ensure the quality of the surveillance of AMR programme, regular feedback, refresher training, technical support, updating of SOPs, dissemination workshops and motivation of the laboratory staff are vital. Programme planning should pay attention to data collection, compilation and storage; an electronic data collection system and appropriate data backup system can reduce the workload of manual/semi electronic labour-intensive system. The surveillance of AMR has continued in Nepal with minimal external financial support; financial dependency on donors should be eliminated. The expansion of laboratory network, gradual incorporation of other pathogens as per local need and resistance testing for additional antimicrobials may be necessary. Analysis and timely feedback of surveillance of AMR data and findings can ensure the use of the data to guide local practices. Attention should be paid to human resources, training, standardisation, capacity building and ensuring the ownership of the programme (isolates and data) by the participating laboratories to ensure sustainability.

## Abbreviations

AMR: Antimicrobial resistance; MOH: Ministry of Health; ICDDR B: International Centre for Diarrhoeal Disease Research, Bangladesh, Dhaka; NPHL: National Public Health Laboratory; EQA: External quality assurance; WHO: World Health Organisation; USAID: United States’ Agency for International Development; RPM: Rational Pharmaceutical Management Project; USCDC: United States’ Center for Disease Prevetion and Control; MDR: Multiple drug resistant; EDCD: Epidemiology and Disease Control Division; MIC: Minimum Inhibitory Concentration; ATCC: American type culture collection; QC: Quality control; STD: Sexually transmitted disease; LZH: Lumbini Zonal Hospital; DH: Dhulikhel Hospital.

## Competing interests

The authors declare that they have no competing interests.

## Authors’ contributions

SM: AMR programme planning, implementation, data analysis, manuscript review. GS: AMR programme planning, implementation, data analysis, manuscript review. SPD: AMR programme, implementation, data analysis. PK: AMR programme implementation, data analysis. BR: AMR programme implementation, data analysis. AH: AMR programme planning, coordination, implementation, data analysis, manuscript review. GBN: AMR programme planning and coordination, data analysis, manuscript review. MJA: AMR programme planning and coordination, data analysis, manuscript review. DS: AMR programme planning, securing funding, donor coordination, implementation monitoring, data analysis, manuscript review. SB: Data analysis, manuscript review. MR: AMR programme planning, coordination and implementation, data analysis, manuscript drafting and manuscript review. All authors read and approved the final manuscript.

## Authors’ information

Antimicrobial Susceptibility Surveillance Programme team, Nepal and participating laboratories. i) Dr. Sarala Malla, Dr. Geeta Shakya and Mr. Shyam P Dumre, NPHL, Kathmandu, ii) Dr. Chandrika Devi Shrestha and Ms. Jyotsna Shrestha, Bir Hospital Laboratory, Kathmandu, iii) Mr. Ram Babu Shrestha and Ms. Sabina Dangol, Patan Hospital Laboratory, Kathmandu, iv) Dr. T. P. Rajbhandari and Ms. Maiya Shrestha, Kanti Children’s Hospital Laboratory, Kathmandu, v) Mr. Nuchchhe Ranta Tuladhar and Mr. Nabaraj Banjade, Tribhuvan University Teaching Hospital Laboratory, Kathmandu, vi) Dr. Syamal Bhattacharya and Dr. Basudha Khanal, B. P. Koirala Institute of Health Sciences, Dahran, vii) Mr. Jagat Khadka and Mr. Boj Bdr Sunuwar, Western Regional Hospital Laboratory, Pokhara, viii) Dr. P.G. Shivananda and Dr. Joshy M Eason, Manipal Teaching Hospital Laboratory, Pokhara, ix) Mr. Hem Bdr Nepali, United Mission Hospital Laboratory, Palpa, x) Mr. Binod Kumar Gyawali, Lumbini Zonal Hospital, Butwal, xi) Mr. Ganesh Neupane, Dhulikhel Hospital, Kabhre and EDCD/Ministry of Health and Population.

## Pre-publication history

The pre-publication history for this paper can be accessed here:

http://www.biomedcentral.com/1471-2458/14/269/prepub
